# Dr. William J. Squire

**Published:** 1917-06

**Authors:** 


**Dr. William J. Squire,** of Buffalo, 1893, died April 28, aged 57.

